# Uncivil customers and work-family spillover: examining the buffering role of ethical leadership

**DOI:** 10.1186/s40359-025-02944-1

**Published:** 2025-07-03

**Authors:** Jeeyoon Jeong, Ji Hoon Lee, Hoora Hajian Karahroodi, Insik Jeong

**Affiliations:** 1 Nagoya University of Commerce and Business, Nisshin, Japan; 2 Southern Illinois University Carbondale, Carbondale, USA; 3 Korea University Business School, Seoul, Korea, Republic of

**Keywords:** Customer incivility, Burnout, Work-family conflict, Ethical leadership

## Abstract

**Background:**

This study investigates the impact of customer incivility on employee burnout and work-family conflict, examining the moderating role of ethical leadership in these relationships within the service industry. By integrating the Conservation of Resources theory and Role Theory, we seek to provide a more comprehensive understanding of how negative customer interactions affect employee well-being beyond the immediate work setting.

**Methods:**

We conducted a two-wave, time-lagged survey among 586 full-time service sector employees in South Korea. The data were analyzed using confirmatory factor analysis and hierarchical regression to test the hypothesized relationships between customer incivility, burnout, work-family conflict, and ethical leadership.

**Results:**

Our results reveal a positive relationship between customer incivility and work-family conflict, which is mediated by burnout. Furthermore, we found that ethical leadership moderates the relationship between customer incivility and burnout, such that the positive relationship is weaker when ethical leadership is high.

**Conclusion:**

By integrating Conservation of Resources theory and Role Theory, this study provides a comprehensive understanding of how negative customer interactions affect employee well-being beyond the immediate work setting. The results highlight the spillover effects of customer incivility on employees’ personal lives and the importance of ethical leadership in mitigating these effects. This research offers valuable insights for developing targeted interventions to enhance employee well-being, work-family balance, and organizational effectiveness in service industries.

## Theoretical background

Customer incivility, characterized by disrespectful and rude customer behaviors, has emerged as a pervasive issue in service-oriented organizations, with frontline employees bearing the brunt of its impact [19, 31, 34, 42]. These uncivil behaviors extend beyond mere isolated incidents, often becoming a chronic source of stress for employees [2]. The repercussions of customer incivility are not limited to the immediate employee-customer interaction,rather, they have far-reaching consequences for both employees and the organization as a whole [46, 49, 50].

While a substantial body of research has investigated the impact of customer incivility on employees’ work-related attitudes and behaviors [19, 46, 49], there remains a notable gap in understanding how this stressor spills over into employees’ personal lives, particularly in the context of work-family conflict [54]. Work-family conflict arises when the demands and responsibilities of work and family roles are mutually incompatible, making it difficult for individuals to fulfill their obligations in both domains [24]. This conflict can have profound consequences that extend beyond the individual employee, affecting the well-being of their families and the overall effectiveness of the organization [6, 14].

The present study aims to address this research gap by exploring the link between customer incivility and work-family conflict, and by investigating the underlying processes that explain this relationship. We draw upon the Conservation of Resources (COR) theory [27] and Role Theory as theoretical frameworks to guide our investigation. COR theory posits that individuals strive to obtain, retain, and protect their valuable resources, and that the threat of resource loss or actual loss of resources leads to stress and strain. In the context of customer incivility, we propose that the chronic exposure to rude and disrespectful customer behaviors leads to a depletion of employees’ emotional and cognitive resources, which in turn contributes to heightened work-family conflict.

Role Theory, on the other hand, suggests that individuals occupy multiple roles in their lives, each with its own set of expectations and demands. When the demands of one role (e.g., work) interfere with the demands of another role (e.g., family), inter-role conflict arises [24]. Integrating COR theory and Role Theory, we argue that customer incivility, as a chronic work stressor, not only depletes employees’ resources but also creates conflicting demands between work and family roles. The resource depletion caused by customer incivility may render employees less capable of meeting the expectations and demands of their family role, thereby intensifying work-family conflict.

Furthermore, we introduce burnout as a mediator in the relationship between customer incivility and work-family conflict. Burnout, characterized by emotional exhaustion, depersonalization, and reduced personal accomplishment [38], is a common consequence of prolonged exposure to work stressors such as customer incivility. Drawing from COR theory, we argue that the resource depletion caused by customer incivility leads to burnout, as employees struggle to cope with the chronic stress. Moreover, from a Role Theory perspective, burnout may further exacerbate the inter-role conflict between work and family. The emotional exhaustion and reduced personal accomplishment associated with burnout may spill over into the family domain, making it more challenging for employees to fulfill their family role expectations [9, 11].

In addition to examining the direct and indirect effects of customer incivility on work-family conflict, we also consider the moderating role of ethical leadership in this relationship. Ethical leadership, which involves the demonstration of normatively appropriate conduct and the promotion of such conduct among followers [18], has been shown to mitigate the negative impact of various workplace stressors. Drawing from COR theory, we conceptualize ethical leadership as a valuable resource that can help employees cope with the demands of customer incivility. Specifically, we posit that ethical leaders, through their supportive and principled behaviors, can buffer the depleting effects of customer incivility on employees’ resources, thereby attenuating the indirect relationship between customer incivility and work-family conflict via burnout.

**Fig. 1:**
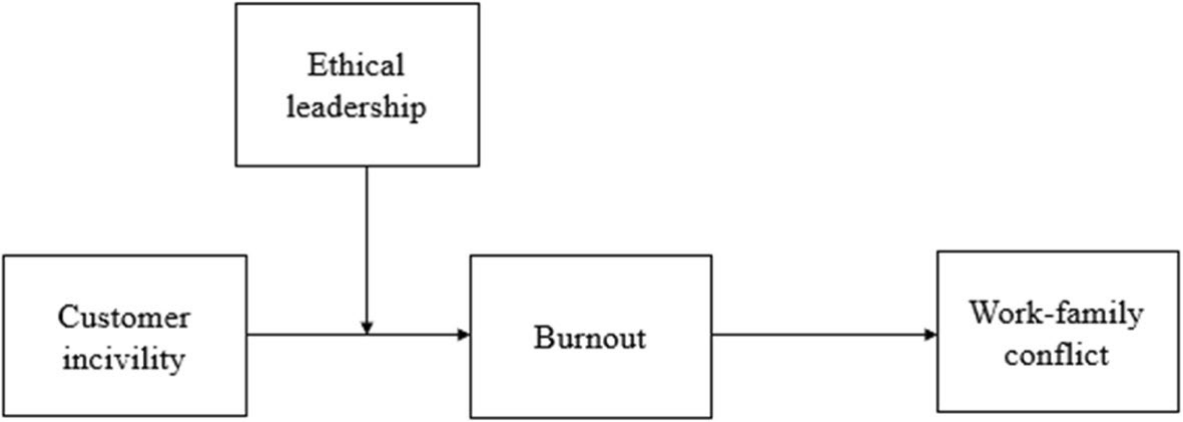
Theoretical model

By investigating the spillover effects of customer incivility on work-family conflict, the mediating role of burnout, and the moderating role of ethical leadership, this study makes several notable contributions to the literature. First, we extend the current understanding of the far-reaching consequences of customer incivility by examining its impact on employees’ non-work domain, specifically work-family conflict. This broadens the scope of customer incivility research beyond the immediate work context and sheds light on the pervasive nature of this stressor. Second, by integrating COR theory and Role Theory, we provide a more comprehensive theoretical framework that elucidates the underlying mechanisms linking customer incivility to work-family conflict. This theoretical integration enhances our understanding of the processes through which customer incivility influences employees’ personal lives and contributes to the advancement of both COR theory and Role Theory in the context of service work. Third, by investigating the moderating role of ethical leadership, we highlight the importance of leadership in mitigating the negative effects of customer incivility and offer valuable insights for organizations aiming to support their employees’ well-being. The conceptual model guiding our study is presented in Fig. 1.

### Theoretical framework

#### Conservation of Resources (COR) theory

The Conservation of Resources (COR) theory, proposed by Hobfoll [27], is a prominent framework for understanding stress and motivation in the workplace. The core tenet of COR theory is that individuals strive to obtain, retain, foster, and protect their valuable resources [28]. Resources are broadly defined as objects, personal characteristics, conditions, or energies that are valued by the individual [27]. COR theory posits that stress occurs when individuals face the threat of resource loss, experience actual resource loss, or fail to gain resources following significant resource investment [29, 31]. The theory emphasizes the importance of resource investment and conservation strategies [26]. In the context of work, COR theory has been applied to explain a wide range of phenomena, including job stress, burnout, work-family conflict, and employee well-being [26, 28].

Recent developments in COR theory have expanded its application in organizational contexts. Hobfoll et al. [28] emphasized the importance of resource caravans and resource passageways in understanding how resources operate within organizational settings. Resource caravans refer to the tendency of resources to aggregate and travel together, while resource passageways are the environmental conditions that support, foster, enrich, or protect these resource caravans [13]. In the context of our study, ethical leadership can be conceptualized as creating resource passageways that enable employees to better manage their resources when faced with customer incivility.

Additionally, recent research has highlighted the dynamic nature of resource loss and gain spirals. When employees experience customer incivility, they may enter a resource loss spiral where the initial resource depletion makes them more vulnerable to further resource loss [52], including increased burnout and work-family conflict. Conversely, supportive leadership can initiate resource gain spirals, where initial resource gains facilitate subsequent acquisition of additional resources [36].

### Role theory

Role Theory is an influential framework that has been widely used to understand the multiple roles that individuals occupy in their lives and the conflicts that can arise between these roles [12];. According to Role Theory, individuals hold various social positions, each associated with a set of expected behaviors or roles [12]. These roles can be related to work or non-work domains and are often defined by social norms, expectations, and the individual’s own understanding of what the role entails [30]. A key concept in Role Theory is role conflict, which occurs when an individual experiences incompatible expectations or demands from different roles. Work-family conflict, a specific form of inter-role conflict, arises when the demands of work and family roles are mutually incompatible [24]. Role Theory also highlights the importance of role stressors, such as role ambiguity, role overload, and role conflict, in influencing individuals’ well-being and performance [45].

By integrating COR theory and Role Theory, we aim to provide a more comprehensive understanding of the mechanisms linking customer incivility to work-family conflict. COR theory helps us understand how customer incivility leads to resource depletion and burnout, while Role Theory illuminates how resource depletion can exacerbate inter-role conflict between work and family domains. Together, these theories provide a solid foundation for our hypothesized relationships.

### Theories and hypotheses

#### Customer incivility and work-family conflict

Drawing from the Conservation of Resources (COR) theory [27] and Role Theory, we propose that customer incivility is positively related to work-family conflict. COR theory posits that individuals strive to obtain, retain, foster, and protect resources they value [28]. When faced with the threat of resource loss or actual resource loss, individuals experience stress and strain [28]. In the context of service work, customer incivility represents a significant job demand and a threat to resources [19]. Dealing with rude, disrespectful, or hostile customers requires emotional and cognitive resources, such as self-control, emotional regulation, and problem-solving skills [11]. Over time, the chronic exposure to customer incivility can lead to resource depletion, as employees struggle to cope with the ongoing demands of their job [19].

Role Theory suggests that individuals occupy multiple roles in their lives, each with its own set of expectations and demands [12]. When the demands of one role (e.g., work) interfere with the ability to fulfill the demands of another role (e.g., family), inter-role conflict arises [24]. Work-family conflict, a form of inter-role conflict, occurs when the demands of work and family roles are mutually incompatible [24]. The resource depletion caused by customer incivility, as conceptualized by COR theory, can make it more difficult for employees to meet the expectations and demands of their family role [6]. This can lead to a sense of imbalance between work and family roles, as employees struggle to fulfill their family responsibilities while coping with the stress of their job [40].

Empirical research has provided support for the link between customer incivility and work-family conflict. For example, Zhu et al. [54] found that customer incivility was positively related to employees’ family undermining behavior, suggesting that the stress of dealing with uncivil customers can spill over into the family domain. Similarly, Greenbaum et al. [25] found that customer mistreatment was positively associated with work-family conflict, and that this relationship was mediated by emotional exhaustion. These empirical findings, along with others in the literature (e.g., [6], provide strong support for the theoretical arguments linking customer incivility to work-family conflict through the mechanisms of resource depletion and inter-role conflict. Based on the theoretical arguments and empirical evidence presented above, we hypothesize the following:

Hypothesis 1 Customer incivility will be positively related to work-family conflict.

### The mediating role of burnout

Building upon the Conservation of Resources (COR) theory [27] and Role Theory, we propose that burnout mediates the relationship between customer incivility and work-family conflict. Burnout is a psychological syndrome characterized by emotional exhaustion, depersonalization, and reduced personal accomplishment that can occur among individuals who work with people in some capacity [38].

From a COR theory perspective, burnout can be conceptualized as a state of resource depletion that occurs when individuals are confronted with chronic job demands that exceed their available resources [28]. Customer incivility, as a chronic job demand, requires service employees to expend significant emotional and cognitive resources to maintain a professional demeanor and to provide high-quality service (Bavik et al., 2021). Over time, the constant need to regulate one’s emotions and to deal with difficult customers can lead to burnout, as employees feel drained and depleted of their resources [23].

Role Theory suggests that burnout can have significant spillover effects on individuals’ ability to fulfill their roles in other domains, such as family. When employees are experiencing burnout, they may have fewer emotional and cognitive resources available to invest in their family role [6]. This can lead to a sense of inter-role conflict, as employees struggle to meet the demands of their family role while coping with the exhaustion and detachment of burnout [6].

Empirical research has provided support for the mediating role of burnout in the relationship between job demands and work-family conflict. For example, Travis et al. [48] found that job burnout mediated the relationship between various job demands and work-family conflict. Similarly, Zhu et al. [54] found that emotional exhaustion mediated the relationship between customer incivility and family undermining among frontline employees in the hospitality industry. Moreover, research has highlighted the importance of considering the multidimensional nature of burnout in understanding its effects on work-family conflict [15]. Based on the theoretical arguments and empirical evidence presented above, we propose the following hypothesis:

Hypothesis 2 Burnout will mediate the relationship between customer incivility and work-family conflict.

### The moderating role of ethical leadership

Extending the Conservation of Resources (COR) theory [27] and integrating it with the literature on ethical leadership [18], we propose that ethical leadership moderates the relationship between customer incivility and burnout. Ethical leadership is defined as “the demonstration of normatively appropriate conduct through personal actions and interpersonal relationships, and the promotion of such conduct to followers through two-way communication, reinforcement, and decision-making” ([18], p. 120). Ethical leaders are characterized by honesty, trustworthiness, fairness, and care, and they act as role models for ethical behavior in the workplace [17, 39].

In this study, we specifically focus on employees’ perceptions of their immediate supervisors’ ethical behaviors, including fairness, integrity, and people-orientation. This approach aligns with Brown et al.’s [18] conceptualization of ethical leadership as being perceived by followers rather than as an objective organizational metric. It is important to note that our study was conducted in South Korea, a cultural context where hierarchical relationships and Confucian values significantly influence leadership perceptions [43]. In Confucian cultures like South Korea, the emphasis on respect for authority, maintaining harmony, and collective welfare may shape how employees interpret and respond to ethical leadership behaviors. For instance, ethical leaders who demonstrate care for employees’ well-being and protect them from customer mistreatment may be particularly valued in this cultural context, as such behaviors align with the Confucian principles of benevolence and righteousness.

From a COR theory perspective, ethical leadership can be conceptualized as a contextual resource that can help employees cope with the demands of their job and protect against resource loss [28]. When faced with customer incivility, service employees may turn to their leaders for support, guidance, and protection. Thus, when ethical leadership is high, by virtue of their caring and supportive nature, employees who mistreated by customers may receive emotional support to employees, thereby replenishing their emotional resources [47]. Moreover, high ethical leadership can create a positive and supportive work environment that buffers against the negative effects of customer incivility (Bani-Melhem et al., 10).

On the other hand, when ethical leadership is low, employees may feel unsupported and unprotected in the face of customer incivility. In the absence of clear guidance and support from their leaders, employees may struggle to cope with the emotional demands of customer mistreatment, leading to greater emotional exhaustion and depersonalization [19]. Moreover, a lack of ethical leadership may create a work environment that is perceived as unfair, unsupportive, and even hostile, exacerbating the negative effects of customer incivility on employee well-being.

Empirical research has provided support for the moderating role of leadership in the relationship between job demands and employee well-being. For example, Anand et al. [7] found that ethical leadership moderated the relationship between workplace incivility and cynicism among frontline service employees. Based on the theoretical arguments and empirical evidence presented above, we propose the following hypothesis:

Hypothesis 3 The positive relationship between customer incivility and burnout will be moderated by ethical leadership. Specifically, when ethical leadership is high, the positive relationship between customer incivility and burnout will be attenuated than when ethical leadership is low.

### Integrated model: moderated-moderated mediation

As shown in Fig. 1, in developing an integrative model that encapsulates the association between customer incivility, burnout, work-family conflict, and ethical leadership, we synthesize the notions that customer incivility is expected to increase work-family conflict via mediating role of burnout and that this mediated relationship is contingent upon the level of ethical leadership present.

Specifically, ethical leadership is proposed to function as a moderating variable in this relationship, providing the resources and support that can alter employees’ perceptions and experiences of incivility, as well as their capacity to cope with its negative effects. In environments where ethical leadership is pronounced, the leaders’ demonstration of ethical behavior, decision-making, and support is likely to provide a buffer against the resource depletion caused by customer incivility, thereby attenuating the mediation effect of burnout on work-family conflict.

Conversely, in environments where ethical leadership is lacking, the absence of this supportive resource is likely to leave employees more susceptible to the influence of customer incivility. As such, the path from customer incivility to burnout and subsequently to work-family conflict may be exacerbated, resulting in a stronger mediated relationship due to the lack of protective resources that ethical leadership would otherwise provide. Therefore, we proposed the following hypothesis:

Hypothesis 4 Ethical leadership will moderate the indirect relationship between customer incivility and work-family conflict via burnout, such that when ethical leadership is high, the indirect relationship will be attenuated than when it is low.

## Materials and methods

### Participants and procedures

This study employed a two-wave, time-lagged survey design to collect data from full-time employees working in the service industry in South Korea. The service industry was chosen as the research context because customer incivility is more prevalent in this sector [46]. The two-wave design was used to reduce common method bias [44] and to capture the development of burnout and changes in work-family conflict over time.

Data were collected through an online survey administered by Macromill Embrain, a leading market research company in South Korea with an extensive panel of service industry employees. The company specializes in online surveys and has a database of over 1.8 million registered panel members across various industries. The specific service company from which participants were recruited was a large retail chain operating multiple department stores and supermarkets throughout South Korea. This context is particularly relevant for our study as retail employees frequently interact with customers and are often exposed to customer incivility.

Participants were informed that their participation was voluntary, and their responses would remain confidential. As an incentive, participants were offered small monetary compensation for completing the surveys. At Time 1, participants responded to questions about customer incivility, ethical leadership, and demographic information. Two weeks later, at Time 2, participants completed measures of burnout and work-family conflict. The two-week time lag was chosen based on previous research suggesting that this time interval is appropriate for examining the effects of job stressors on employee well-being.

Of the 1,042 employees who completed the Time 1 survey, 728 completed the Time 2 survey, yielding a response rate of 69.9%. After excluding participants who failed to pass the attention checks (e.g., “Please select ‘strongly agree’ for this item”), the final sample consisted of 586 employees (56.2% male; average age = 38.42 years, SD = 11.84). The majority of participants were married (60.8%), had a college degree or higher (62.3%), and had been working in their current organization for more than 5 years (58.2%). In terms of position, 62.5% were frontline employees (sales associates, customer service representatives), 28.3% were middle managers (department supervisors, team leaders), and 9.2% were senior managers. The position variable was important in our study as employees in different hierarchical positions may experience varying levels of customer incivility and have different resources to cope with such stressors.

To assess potential non-response bias, we compared the demographic characteristics of participants who completed both surveys (*n* = 586) with those who only completed the Time 1 survey (*n* = 456). There were no significant differences in gender, age, marital status, education level, or tenure between the two groups, suggesting that non-response bias was not a significant concern in this study.

### Measurement

All survey instruments were subjected to a rigorous back-translation procedure [16] to ensure their linguistic and conceptual equivalence. All items were rated on a 5-point Likert-type scale, ranging from 1 (strongly disagree) to 5 (strongly agree).

Customer incivility was measured at Time 1 using a five-item scale developed by Greenbaum et al. [25]. This scale assesses employees’ experiences of disrespectful and rude behaviors from customers. A sample item is: “Customers direct offensive remarks towards me” (Cronbach’s α = 0.85). Ethical leadership was also measured at Time 1 using a 10-item scale developed by Brown et al. [18]. Sample items are: “He/she sets an example of how to do things the right way in terms of ethics” and “He/she discusses business ethics or values with employees” (Cronbach’s α = 0.90). Burnout was assessed at Time 2 using the 4-item work-related burnout dimension of the Copenhagen Burnout Inventory (CBI; [21, 33]). Sample items included “I feel emotionally exhausted at work” and “I feel burned out from my work” (Cronbach’s α = 0.88). Work-family conflict was measured at Time 2 using a five-item scale developed by Netemeyer et al. [41]. A sample item is: “The demands of my work interfere with my home and family life” (Cronbach’s α = 0.90).

We controlled for several demographic variables that have been shown to influence work-family conflict and burnout in previous research. Age was controlled because older employees may have developed better coping strategies for dealing with work stressors over time [4]. Gender was included as a control variable because research suggests that men and women may experience and cope with work-family conflict differently [5]. Organizational tenure was controlled because employees with longer tenure may have developed stronger support networks within the organization, potentially buffering the negative effects of customer incivility [29]. Education level was controlled because higher levels of education might be associated with greater access to resources and coping strategies [40]. Finally, we controlled for position within the organization, as employees in higher positions may have more autonomy and resources to deal with customer incivility [32].

### Statistical analysis

To test our hypotheses, we conducted several analyses. First, we performed a confirmatory factor analysis (CFA) to assess the distinctiveness of our study variables and to evaluate the measurement model. Second, to mitigate common method bias, we employed several procedural and statistical remedies. Procedurally, we used a time-lagged design, protected respondents’ anonymity, and used different response formats for different measures. Statistically, we conducted Harman’s single-factor test and used the common latent factor method to assess the potential influence of common method variance. Third, we used hierarchical regression analysis to test our hypotheses regarding the direct relationships between customer incivility and work-family conflict, as well as the moderating role of ethical leadership in the relationship between customer incivility and burnout. Finally, we employed bootstrapping procedures to test the mediation and moderated mediation hypotheses.

## Results

### Descriptive statistics

Table 1 displays descriptive statistics, encompassing averages and standard deviations for the variables in the study. The correlation analysis revealed a positive correlation between customer rudeness and burnout (*r* = 0.43, *p* < 0.01), and a notably positive relationship with work-family conflict (*r* = 0.38, *p* < 0.01). Furthermore, a significant positive relationship was found between burnout and work-family conflict (*r* = 0.51, *p* < 0.01).

**Table 1:** Descriptive statistics, correlations, and reliabilities among variables

	M	SD	1	2	3	4	5	6	7
1.Age	38.42	11.84	–						
2.Gender ^a^	.44	.50	–.05	–					
3.Education	1.96	.78	–.01	.05	–				
4.Tenure	11.06	5.40	.04	.12*	.08	–			
5.Customer incivility	2.98	1.16	–.04	.05	.11*	.01	–		
6.Ethical leadership	3.24	1.10	.08	.08	.04	–.01	–.24***	–	
7.Burnout	3.89	1.17	–.10*	–.06	.03	–.04	.43***	–.29***	–
8.Work-family conflict	3.01	1.23	-.05	.07	–.12*	–.09*	.38***	–.11*	.51***

*N* = 586

^a^Gender was dummy coded (female = 1, male = 0)

^*^p <.05

^**^p <.01

^***^p <.001

### Measurement model

A confirmatory factor analysis (CFA) was conducted to verify the distinctiveness of the study’s constructs. The proposed four-factor model demonstrated an excellent fit, as indicated by the fit indices: χ^2^ (327) = 772.44, CFI = 0.94, TLI = 0.93, RMSEA = 0.07. This model’s fit was superior compared to alternative models, including three-factor, two-factor, and one-factor models, as detailed in Table 2, validating our theoretical model. Additionally, CFA confirmed the convergent validity of the latent constructs, as detailed in Table 3. All constructs showcased construct reliability (CR) values above the benchmark of 0.7, indicating strong reliability. Additionally, the average variance extracted (AVE) for each construct exceeded 0.5, thus affirming their robust convergent validity. This outcome solidifies the presence of a significant degree of convergent validity among the variables in this research.

**Table 2:** Results for measurement model comparisons

**Model**	χ^2^ ***(df)***	**CFI**	**TLI**	**RMSEA**	△χ^2^ ***(***△***df)***
Theoretical 4-factor model (CI, BU, EL, WF)	772.44 (327)	0.94	0.93	0.07	
3-factor model (CI & BU merged, EL, WF)	1038.74 (330)	0.85	0.85	0.12	266.30 (3)**
2-factor model (CI, BU, & EL merged, WF)	1529.01 (332)	0.64	0.61	0.23	756.57 (5)**
1-factor model	2073.48 (333)	0.49	0.48	0.29	1301.04 (6)**

*CI* customer incivility, *BU* burnout, *EL* ethical leadership, *WF* work-family conflict, *CFI* comparative fit index, *TLI* Tucker-Lewis index, *RMSEA* root mean square error of approximation

^**^p < 0.01

**Table 3:** Item measurement properties

Variable		Cronbach alpha	Estimates	AVE	CR	Variance Explained (%)	Cumulative Variance (%)
Customer Incivility	CI1: Customers direct offensive remarks toward me	.85	.81	.68	.85	46.33	46.33
	CI2: Customers treat me in a discourteous manner		.76				
	CI3: Customers make insulting comments to me		.70				
	CI4: Customers ignore me		.72				
	CI5: Customers show a lack of respect for me		.75				
Ethical leadership	EL1: My supervisor sets an example of how to do things the right way in terms of ethics	.90	.92	.72	.90	55.91	102.24
	EL2: My supervisor conducts his/her personal life in an ethical manner		.79				
	EL3: My supervisor listens to what employees have to say		.84				
	EL4: My supervisor disciplines employees who violate ethical standards		.91				
	EL5: My supervisor defines success not just by results but also the way they are obtained		.88				
	EL6: My supervisor discusses business ethics or values with employees		.82				
	EL7: My supervisor makes fair and balanced decisions		.81				
	EL8: My supervisor can be trusted		.78				
	EL9: My supervisor has the best interests of employees in mind		.90				
	EL10: My supervisor asks “what is the right thing to do?” when making decisions		.91				
Burnout	BO1: I feel emotionally drained from my work	.88	.85	.71	.89	51.02	153.26
	BO2: I have become more cynical about whether my work contributes anything		.78				
	BO3: I doubt the significance of my work		.79				
	BO4: I feel burned out from my work		.88				
Work-family Conflict	WF1: The demands of my work interfere with my home and family life	.90	.82	.79	.92	50.24	203.50
	WF2: The amount of time my job takes up makes it difficult to fulfill family responsibilities		.82				
	WF3: Things I want to do at home do not get done because of the demands my job puts on me		.78				
	WF4: My job produces strain that makes it difficult to fulfill family duties		.76				
	WF5: Due to work-related duties, I have to make changes to my plans for family activitieies		.77				

To mitigate concerns related to common method bias, we implemented several procedural and statistical remedies. Procedurally, we used a time-lagged design with a two-week interval between the measurement of independent and dependent variables. Additionally, we assured participants of the anonymity of their responses and varied the response formats for different measures. Statistically, we conducted Harman’s single-factor test. The results demonstrated that the largest factor explained only 24.68% of the total variance, significantly below the 50% threshold, reducing the likelihood that common method bias substantially influenced the results.

### Hypothesis tests

We conducted hierarchical regression analysis to test the research hypotheses. First, Hypothesis 1 assumed that the association between customer incivility and work-family conflict would be positive. After controlling for age, gender, organizational tenure, and education, a notable positive impact of leader narcissism on work-family conflict was discovered in Model 4 of Table 4 (β = 0.52, *p* < 0.01), supporting Hypothesis 1.

**Table 4:** The results of hierarchical regression analysis

	Outcome: Burnout			Outcome: Work-family conflict	
Predictors	Model 1	Model 2	Model 3	Model 4	Model 5
Constant	2.05**	1.96**	1.92***	3.50**	1.42**
Age	–.01	–.01	–.01	–.02*	–.02*
Gender	.05	.06	.04	.00	.01
Education	.10**	.09**	.08*	.15**	.13**
Organizational tenure	.03	.02	.02	–.05	–.04
Customer incivility (A)	.45**	.40**	.35**	.52**	.48**
Ethical leadership (B)		–.25**	–.20**	–.25**	–.20**
A × B			–.15**		–.07
Burnout					.51**
*R*^2^	.20**	.28**	.30**	.23**	.41**
∆*R*^2^		.08**	.02*		.18**

*N* = 586

^*^p <.05, **p <.01

Hypothesis 2 assumed that the impact of customer incivility on work-family conflict would be mediated by burnout. The results showed that customer incivility had a significant positive correlation with burnout. (β = 0.45, *p* < 0.01) in Model 2 of Table 4. Model 5 further demonstrated a substantial positive link between burnout and work-family conflict (β = 0.51, *p* < 0.01). Bootstrapping with 10,000 resamples verified a total effect of customer incivility on work-family conflict at 0.40 (SE = 0.06, 95% CI [0.28, 0.52]), a direct effect of 0.18 (SE = 0.04, 95% CI [0.10, 0.26]), and an indirect effect of 0.22 (SE = 0.03, 95% CI [0.16, 0.28]) even when including burnout in the model. Therefore, the significant indirect effect via burnout (effect = 0.22, SE = 0.03, 95% CI [0.17, 0.27]) supports Hypothesis 2. As the direct effect of customer incivility on work-family conflict remained significant after including burnout in the model, our results indicate that burnout partially mediates the relationship between customer incivility and work-family conflict.

Hypothesis 3 proposed ethical leadership as a moderator in the positive impact of customer incivility on employee burnout. A significant interaction was found in Model 3 of Table 4 (β = −0.15, *p* < 0.01). To create the interaction term, we first standardized the customer incivility and ethical leadership variables to reduce multicollinearity concerns, and then multiplied these standardized variables to create the interaction term [3]. Simple slopes analysis showed that at higher levels of ethical leadership (+ 1 SD), the positive impact of customer incivility on burnout was more attenuated (β = 0.24, SE = 0.02, *p* < 0.01) than when it was low (−1 SD; β = 0.41, SE = 0.04, *p* < 0.01), confirming Hypothesis 3.

We also examined whether ethical leadership moderates the relationship between customer incivility and work-family conflict. The results, however, did not reveal a significant interaction effect (β = −0.07, SE = 0.05, *p* > 0.05), suggesting that ethical leadership specifically buffers the relationship between customer incivility and burnout, rather than directly moderating the relationship between customer incivility and work-family conflict. This finding aligns with our theoretical framework, which posits that ethical leadership primarily functions as a resource that helps employees cope with the immediate stressor (customer incivility) rather than directly affecting the spillover from work to family domains.

Finally, for Hypothesis 4, the conditional indirect effect was evaluated using a bootstrapping approach with 10,000 samples. As illustrated in Table 5, the results indicated a positive indirect effect of customer incivility on work-family conflict via burnout was weakened under higher ethical leadership (indirect effect = 0.18, SE = 0.03, 95% CI [0.09, 0.15]) compared to lower ethical leadership (indirect effect = 0.39, SE = 0.05, 95% CI [0.17, 0.31]). This supports the moderated mediation model proposed in Hypothesis 4.

**Table 5:** The results of conditional indirect effects

	Conditional indirect effects		
Ethical leadership	Indirect effects	Boot SE	Boot 95% CI
Low (mean – 1SD)	.39	.05	[.17.31]
High (mean + 1SD)	.18	.03	[.09,.15]

*N* 586, *Bootstrap N* 10000

## Discussion

### Theoretical implications

The present study makes several noteworthy contributions to the literature on customer incivility, burnout, work-family conflict, and ethical leadership. First, our findings extend the understanding of the far-reaching consequences of customer incivility by examining its impact on employees’ non-work domain, specifically work-family conflict. While previous research has primarily focused on the effects of customer incivility on work-related outcomes, such as job satisfaction, job performance, and turnover intentions [2, 46], our study reveals that the negative impact of customer incivility can spill over into employees’ personal lives, disrupting the work-family interface. This finding highlights the need for researchers to adopt a more holistic perspective when investigating the consequences of customer mistreatment, considering not only the work domain but also the non-work domain.

Second, our study advances the theoretical understanding of the mechanisms linking customer incivility to work-family conflict by integrating the Conservation of Resources (COR) theory [27] and the Role Theory. While previous studies have applied these theories separately to understand the effects of job demands on employee well-being (e.g., 1, 37] our study demonstrates the value of integrating these theoretical perspectives to provide a more comprehensive explanation of the processes underlying the relationship between customer incivility and work-family conflict. Specifically we argue that customer incivility depletes employees’ emotional resources leading to burnout which in turn spills over into the family domain creating inter-role conflict. This theoretical integration extends the COR theory by highlighting the spillover effects of resource depletion across life domains and enriches the Role Theory by identifying burnout as a key mechanism through which job demands can generate inter-role conflict.

Third, our study sheds light on the moderating role of ethical leadership in the relationship between customer incivility and burnout, addressing a significant gap in the literature. While previous research has recognized the buffering effects of various resources, such as social support, autonomy, and self-efficacy, on the relationship between job demands and burnout [39, 51], the role of leadership, particularly ethical leadership, has been largely overlooked [34]. Our findings suggest that ethical leadership can serve as a contextual resource that mitigates the negative impact of customer incivility on employee burnout. This finding extends the COR theory by identifying ethical leadership as a valuable resource that can offset the resource depletion caused by customer incivility and highlights the importance of considering leadership factors in the study of job demands and employee well-being.

Fourth, our study contributes to the growing literature on the dark side of customer behavior [53, 54] by providing empirical evidence for the detrimental effects of customer incivility on service employees’ well-being and work-family balance. While previous research in this area has primarily focused on the consequences of extreme forms of customer mistreatment, such as aggression and violence [29, 31] our study shows that even low-intensity deviant behaviors, such as rudeness and disrespect, can have significant negative impacts on employees. This finding underscores the need for service organizations to take proactive measures to prevent and manage customer incivility, as it can have far-reaching consequences for employee well-being and organizational effectiveness.

Finally, our study contributes to the literature on work-family conflict by identifying customer incivility as a novel antecedent and burnout as a mediating mechanism. While previous research has examined the effects of various job demands, such as workload, role conflict, and time pressure, on work-family conflict [40], the role of customer-related stressors, particularly customer incivility, has been largely overlooked [34]. Our findings suggest that customer incivility can be a significant source of work-family conflict, as it depletes employees’ emotional resources and increases their vulnerability to burnout. This finding extends the nomological network of work-family conflict and highlights the need for researchers to consider customer-related factors when investigating the antecedents of work-family conflict in service settings.

### Practical implications

Our findings offer several practical implications for service organizations seeking to mitigate the negative impact of customer incivility on employee well-being and work-family balance. First, given the detrimental effects of customer incivility on employee burnout and work-family conflict, service organizations should prioritize the development and implementation of policies and practices aimed at preventing and managing uncivil customer behavior [49]. This could include training programs that help employees identify and respond effectively to customer incivility, such as emotion regulation techniques and assertive communication skills. Organizations might also consider implementing clear protocols for handling difficult customers, including escalation procedures when customer behavior becomes particularly problematic. Additionally, creating a supportive organizational climate where employees feel comfortable reporting incidents of customer incivility can help management address systemic issues and protect employees from repeated exposure to such stressors.

Second, our findings underscore the critical role of ethical leadership in mitigating the negative impact of customer incivility on employee well-being. Service organizations should focus on developing and promoting ethical leadership practices at all levels of management [8]. This could involve providing leadership training programs that emphasize ethical decision-making, fairness, integrity, and employee well-being. Organizations might establish formal mentoring structures where experienced leaders model ethical behaviors for newer managers. Additionally, leadership development programs could specifically address how to support employees who face customer incivility, including techniques for intervention, providing emotional support, and creating a protective buffer between difficult customers and frontline staff. Organizations should also consider including ethical leadership behaviors in performance evaluations and promotion criteria to reinforce their importance.

Third, our study highlights the importance of addressing burnout among service employees. Service organizations should implement interventions aimed at preventing and reducing employee burnout, such as providing employees with resources and support to cope with job demands, regularly assessing and monitoring employee burnout levels, and taking proactive steps to address any identified issues [9]. Such interventions might include workload management initiatives, stress reduction programs, and resilience training. Organizations could also implement regular “pulse surveys” to monitor burnout levels and identify departments or teams where additional support is needed. Employee assistance programs offering counseling services and wellness resources can provide additional support for employees experiencing high levels of stress or burnout.

Fourth, service organizations should recognize the spillover effects of work stress on employees’ non-work lives and take steps to support work-family balance. This could involve implementing family-friendly policies and practices, training managers to be sensitive to employees’ work-family needs, and creating a work environment that values and supports work-family balance [4, 35]. Specific policies might include flexible work arrangements, adequate paid time off, and clear boundaries around after-hours communication. Organizations could also offer resources such as childcare assistance, elder care referrals, or other family support services that help employees manage their dual responsibilities. Additionally, creating a culture where utilizing family-friendly benefits is encouraged rather than stigmatized is crucial for their effectiveness.

Fifth, our findings suggest that service organizations should take a holistic approach to employee well-being, considering both work-related and non-work factors that may impact employee health and performance [22]. This could involve implementing wellness programs, providing resources and support for stress management and resilience building, and fostering a culture that promotes employee well-being. Such programs might include stress management workshops, mindfulness training, physical fitness initiatives, and activities that foster social connection and community among employees. Organizations should also consider the role of job design in promoting well-being, such as ensuring appropriate autonomy, skill variety, and opportunities for recovery during the workday.

Finally, our study highlights the need for service organizations to regularly assess and address the impact of customer incivility on employee well-being and work-family balance [54]. This could involve conducting periodic surveys, focus groups, or interviews to understand employees’ experiences with customer incivility and its effects on their work and personal lives. Organizations should use this information to refine their policies, training programs, and support systems to better protect employees from the negative consequences of customer incivility. Additionally, involving employees in developing solutions can lead to more effective interventions and greater employee buy-in.

### Limitations and future directions

While this study makes significant contributions to the literature on customer incivility, burnout, work-family conflict, and ethical leadership, it is important to acknowledge its limitations and provide directions for future research. First, our study relied on self-report measures from a single source, which may raise concerns about common method bias [44]. Future research could benefit from using multiple sources of data to provide a more objective assessment of these constructs. Second, our study focused on a sample of full-time service employees in South Korea, which may limit the generalizability of our findings to part-time employees or those in other cultural contexts. Future research should examine these relationships in different cultural settings and across various employment types to assess the generalizability of our findings [31]. Third, while our study controlled for several demographic variables, we did not account for other potentially relevant factors, such as employees’ personality traits, coping strategies, or family characteristics [40]. Future research could examine how these factors may moderate the relationships among customer incivility, burnout, and work-family conflict. Fourth, our study employed a two-wave design with a two-week time lag, which may not fully capture the dynamic nature of these constructs over time [46]. Future research could use experience sampling methods or daily diary studies to examine how these constructs may vary within individuals over shorter time intervals. Finally, our study focused on the negative consequences of customer incivility for employee well-being and work-family balance, but it did not examine how these outcomes may subsequently impact organizational effectiveness or customer satisfaction. Future research could explore the broader organizational implications of customer incivility [20].

## Data Availability

The datasets in this research are available from the corresponding author on reasonable request.

## References

[1] Agolli A, Holtz BC. Facilitating detachment from work: A systematic review, evidence-based recommendations, and guide for future research. J Occup Health Psychol. 2023;28(3):129–59. 37141024 10.1037/ocp0000353

[2] Al-Hawari MA, Bani-Melhem S, Quratulain S. Do frontline employees cope effectively with abusive supervision and customer incivility? Testing the effect of employee resilience. J Bus Psychol. 2020;35(2):223–40.

[3] Aiken LS, West SG, Reno RR. Multiple regression: Testing and interpreting interactions. Sage; 1991.

[4] Allen TD, French KA, Dumani S, Shockley KM. Meta-analysis of work–family conflict mean differences: Does national context matter? J Vocat Behav. 2015;90:90–100.

[5] Allen TD, Johnson RC, Saboe KN, Cho E, Dumani S, Evans S. Dispositional variables and work–family conflict: A meta-analysis. J Vocat Behav. 2012;80(1):17–26.

[6] Amstad FT, Meier LL, Fasel U, Elfering A, Semmer NK. A meta-analysis of work–family conflict and various outcomes with a special emphasis on cross-domain versus matching-domain relations. J Occup Health Psychol. 2011;16(2):151–69. 21280939 10.1037/a0022170

[7] Anand A, Agarwal UA, Offergelt F. Why should I let them know? Effects of workplace incivility and cynicism on employee knowledge hiding behavior under the control of ethical leadership. Int J Manpow. 2023;44(2):247–66.

[8] Babalola MT, Stouten J, Camps J, Euwema M. When do ethical leaders become less effective? The moderating role of perceived leader ethical conviction on employee discretionary reactions to ethical leadership. J Bus Ethics. 2019;154:85–102.

[9] Bakker AB, Demerouti E. Job demands–resources theory: Taking stock and looking forward. J Occup Health Psychol. 2017;22(3):273–85. 27732008 10.1037/ocp0000056

[10] Bani-Melhem S. What mitigate and exacerbate the influences of customer incivility on frontline employee extra-role behaviour? J Hospitality Tourism Management. 2020:44:38-49.

[11] Bavik A, Bavik YL. Effect of employee incivility on customer retaliation through psychological contract breach: The moderating role of moral identity. Int J Hosp Manag. 2015;50:66–76.

[12] Biddle BJ. Recent developments in role theory. Annu Rev Sociol. 1986;12(1):67–92.

[13] Boley BB. Conservation of Resources Theory: A new theory for the resident attitude literature. Ann Tour Res. 2025;112:103949.

[14] Bolino MC, Turnley WH. The personal costs of citizenship behavior: the relationship between individual initiative and role overload, job stress, and work-family conflict. J Appl Psychol. 2005;90(4):740–8. 16060790 10.1037/0021-9010.90.4.740

[15] Bourhis A, Mekkaoui R. Beyond work-family balance: Are family-friendly organizations more attractive? Relat Ind. 2010;65(1):98–117.

[16] Brislin RW. Cross-cultural research methods: Strategies, problems, applications. In: Environment and culture. Boston: Springer US; 1980:47–82.

[17] Brown ME, Treviño LK. Ethical leadership: A review and future directions. Leadersh Q. 2006;17(6):595–616.

[18] Brown ME, Treviño LK, Harrison DA. Ethical leadership: A social learning perspective for construct development and testing. Organ Behav Hum Decis Process. 2005;97(2):117–34.

[19] Cheng B, Dong Y, Zhou X, Guo G, Peng Y. Does customer incivility undermine employees’ service performance? Int J Hosp Manag. 2020;89:102544.

[20] Choi HJ, Kim YT, Cho YC. The effect of job stress and job satisfaction on burnout of caregivers in geriatric hospitals. Korean J Occup Health Nurs. 2021;30(1):10–9.

[21] Fastje F, Mesmer-Magnus J, Guidice R, Andrews MC. Employee burnout: the dark side of performance-driven work climates. J Organ Eff People Perform. 2023;10(1):1–21.

[22] Fleming WJ. Employee well-being outcomes from individual-level mental health interventions: Cross-sectional evidence from the United Kingdom. Ind Relat J. 2024;55(2):162–82.

[23] Grandey AA, Melloy RC. The state of the heart: Emotional labor as emotion regulation reviewed and revised. J Occup Health Psychol. 2017;22(3):407–22. 28150996 10.1037/ocp0000067

[24] Greenhaus JH, Beutell NJ. Sources of conflict between work and family roles. Acad Manag Rev. 1985;10(1):76–88.

[25] Greenbaum RL, Quade MJ, Mawritz MB, Kim J, Crosby D. When the customer is unethical: the explanatory role of employee emotional exhaustion onto work–family conflict, relationship conflict with coworkers, and job neglect. J Appl Psychol. 2014;99(6):1188. 24955868 10.1037/a0037221

[26] Halbesleben JR, Neveu JP, Paustian-Underdahl SC, Westman M. Getting to the “COR” understanding the role of resources in conservation of resources theory. J Manag. 2014;40(5):1334–64.

[27] Hobfoll SE. Conservation of resources: a new attempt at conceptualizing stress. Am Psychol. 1989;44(3):513–24. 2648906 10.1037//0003-066x.44.3.513

[28] Hobfoll SE, Halbesleben J, Neveu JP, Westman M. Conservation of resources in the organizational context: The reality of resources and their consequences. Annu Rev Organ Psychol Organ Behav. 2018;5:103–28.

[29] Karatepe OM, Nkendong RA. The relationship between customer-related social stressors and job outcomes: the mediating role of emotional exhaustion. Econ Res-Ekon Istraž. 2014;27(1):414–26.

[30] Katz D, Kahn RL. Organizations and the system concept. Classics Organ Theory. 1978;80(480):27.

[31] Kim H, Qu H. The effects of experienced customer incivility on employees’ behavior toward customers and coworkers. J Hosp Tour Res. 2019;43(1):58–77.

[32] Kim TT, Karatepe OM, Chung UY. Got political skill? The direct and moderating impact of political skill on stress, tension and outcomes in restaurants. Int J Contemp Hosp Manag. 2019;31(3):1367–89.

[33] Kristensen TS, Borritz M, Villadsen E, Christensen KB. The Copenhagen Burnout Inventory: A new tool for the assessment of burnout. Work Stress. 2005;19(3):192–207.

[34] Lages CR, Perez-Vega R, Kadić-Maglajlić S, Borghei-Razavi N. A systematic review and bibliometric analysis of the dark side of customer behavior: An integrative customer incivility framework. J Bus Res. 2023;161:113779.

[35] Las Heras M, Rofcanin Y, Matthijs Bal P, Stollberger J. How do flexibility i-deals relate to work performance? Exploring the roles of family performance and organizational context. J Organ Behav. 2017;38(8):1280–94.

[36] Lin X, Wu CH, Hirst G, Chen ZX, Duan J. Why and when servant leadership spurs followers to speak up: A conservation of resources perspective. J Occup Organ Psychol. 2025;98(1):e12561.

[37] Mansour S, Tremblay DG. Work–family conflict/family–work conflict, job stress, burnout and intention to leave in the hotel industry in Quebec (Canada): moderating role of need for family friendly practices as “resource passageways.” Int J Hum Resour Manag. 2018;29(16):2399–430.

[38] Maslach C. What have we learned about burnout and health? Psychol Health. 2001;16(5):607–11. 22804502 10.1080/08870440108405530

[39] Mayer DM, Kuenzi M, Greenbaum R, Bardes M, Salvador RB. How low does ethical leadership flow? Test of a trickle-down model. Organ Behav Hum Decis Process. 2009;108(1):1–13.

[40] Michel JS, Kotrba LM, Mitchelson JK, Clark MA, Baltes BB. Antecedents of work–family conflict: A meta-analytic review. J Organ Behav. 2011;32(5):689–725.

[41] Netemeyer RG, Boles JS, McMurrian R. Development and validation of work–family conflict and family–work conflict scales. J Appl Psychol. 1996;81(4):400–10.

[42] Ni D, Liu X, Zheng X. Render good for evil? The relationship between customer mistreatment and customer-oriented citizenship behavior. J Bus Res. 2024;170:114349.

[43] Park S, Han SJ, Hwang SJ, Park CK. Comparison of leadership styles in Confucian Asian countries. Hum Resour Dev Int. 2019;22(1):91–100.

[44] Podsakoff PM, MacKenzie SB, Lee JY, Podsakoff NP. Common method biases in behavioral research: a critical review of the literature and recommended remedies. J Appl Psychol. 2003;88(5):879–903. 14516251 10.1037/0021-9010.88.5.879

[45] Schmidt S, Roesler U, Kusserow T, Rau R. Uncertainty in the workplace: examining role ambiguity and role conflict, and their link to depression—a meta-analysis. Eur J Work Organ Psychol. 2014;23(1):91–106.

[46] Sliter M, Jex S, Wolford K, McInnerney J. How rude! Emotional labor as a mediator between customer incivility and employee outcomes. J Occup Health Psychol. 2010;15(4):468–81. 21058859 10.1037/a0020723

[47] Tahir NA, Maqsood M. Impact of workplace bullying on job attitudes: Moderated by ethical leadership. Int J Bus Econ Aff. 2020;5(6):281–99.

[48] Travis DJ, Lizano EL, Mor Barak ME. ‘I’m so stressed!’: A longitudinal model of stress, burnout and engagement among social workers in child welfare settings. Br J Soc Work. 2016;46(4):1076–95. 27559215 10.1093/bjsw/bct205 PMC4986087

[49] Walker DD, Van Jaarsveld DD, Skarlicki DP. Exploring the effects of individual customer incivility encounters on employee incivility: The moderating roles of entity (in) civility and negative affectivity. J Appl Psychol. 2014;99(1):151–61. 24060158 10.1037/a0034350

[50] Wu Y, Groth M, Zhang K, Minbashian A. A meta-analysis of the impact of customer mistreatment on service employees’ affective, attitudinal and behavioral outcomes. J Serv Manag. 2023;34(5):896–940.

[51] Xanthopoulou D, Bakker AB, Dollard MF, Demerouti E, Schaufeli WB, Taris TW, Schreurs PJ. When do job demands particularly predict burnout? The moderating role of job resources. J Manag Psychol. 2007;22(8):766–86.

[52] Zhang Z, Wang R, Shang L, Yin K, Liu G, Gui X. Injustice provokes psychological resources loss: A dual-pathway model of app-worker reactions to customers’ injustice. J Bus Ethics. 2025;197(4):713–38.

[53] Zhou ZE, Meier LL, Spector PE. The spillover effects of coworker, supervisor, and outsider workplace incivility on work-to-family conflict: A weekly diary design. J Organ Behav. 2019;40(9–10):1000–12.

[54] Zhu H, Lyu Y, Ye Y. The impact of customer incivility on employees’ family undermining: A conservation of resources perspective. Asia Pac J Manag. 2021;38(3):1061–83.

